# Who Are Dentists?

**Published:** 1861-09

**Authors:** Wm. A. Pease


### WHO ARE DENTISTS?

BY WM. A. PEASE.

THERE appeared in the July number of the New York Dental Journal an article by Dr. J. Allen, reviewing an article of mine, published in the Dental Register of March preceding. It was headed, Who are Dentists. In the August number of the Register and Cosmos the same article appears as an original contribution; to which, however, has been added a page of original matter. When the article was first read in the N. York Dental Journal, it was thought, although the tone and aim of it, and the manner of quotation were any thing but fair and commendable, that it did not require an answer. But, since it has been received and published as an original contribution by two other dental journals, it is fair to presume that they view it with considerable favor, if they do not endorse its general aim and scope. Perhaps on that account, but more especially on account of his peculiar manner of quoting the article in review, by which injustice is done me, it may be proper to notice some of his quotations and the more important parts of his article.

Every dentist is aware of the ignorance of the public, of the capabilities of dentists; and he is frequently inquired of as to the comparative value of different operations, materials and methods of inserting artificial dentures. To enlighten the public mind as far as practicable, early last winter I concluded to write a series of articles for the popular reader, to be first published in the Register, in order that they might reach dentists, who, if they approved of them, could have them copied, in whole or in part, into their local journals. These articles were designed to be practical, to present prominent, salient points in paragraphs, often but little connected with one another, and lumbered with few conditions or exceptions, that they might be the better remembered. They were designed to show, what the people had a right to expect from dentists, and dentists from the people, the comparative value of different operations, materials, and what might reasonably be expected from mechanical dentistry; to show that there are two classes of dentists, operators and mechanics, and that the person who devotes his attention principally to mechanics will generally follow the bent of his genius and skill, and be less likely to be either skillful or successful at the chair; and, on the other hand, the operator will save more teeth. Thus, in the first article, the prominent fact was stated that previous to 1854 ulcers at the roots of teeth were generally intractable, and the teeth became a nuisance in the mouth, and had to be removed; that since that time the necessity of extracting them no longer exists. This was stated as a prominent fact, to excite attention, and it was unincumbered with the conditions, exceptions, or the percentage of failures it would have been necessary to have given, had the article been written for the profession.

Thus, having given the history of the origin of the articles, I shall now proceed to notice Dr. Allen's review. His first quotation is as follows: "Dentistry as a profession is of American origin. It had its rise in a great public want which nowhere but in the United States *has, or could have* existed; and there is little likelihood that it ever will exist in any other country." The reader will observe that the Doctor omits the words in italics, which are important to the sentence, if not to the sense. He then makes these remarks: "The idea here conveyed is, that teeth do not decay except in America. This is an egregious error." The idea there conveyed is precisely what it says; and had the Doctor been as anxious as he claims, to chronicle facts and discard error, he would not have written as he did. That was the more unnecessary, because, if he had looked back one short month to the number of the Register next preceding (February) he would have found the reasons upon which that paragraph was based stated more in detail. That he must have read the article, there is reason to believe, because he goes back more than a year, and quotes from another article a line that suits his purpose; and he appears to have been industrious in searching for authorities; not to vindicate the truth of history; not to show when dentistry as a profession originated, for, for these he probably does not care a fig; but, apparently, for an ulterior purpose, which will be more and more apparent as this article progresses; when at last it will be seen that the cause for which we are indebted for his review exists, not in the first, but in the third article of the series. Dr. Allen continues his quotations. "The want that gave it birth gave it also a vigorous growth, unexampled in any other profession—for dentistry can not truly be said to date back much earlier than 1840 ; *and as a profession, by which is meant the point of time when dentists felt competent to say to the people that, accidents excepted, there is no longer any need for them to lose their teeth, is of as recent date as* 1854. The Baltimore College had just been established, and it was thought that, by scientific investigation, means would soon be found to preserve the natural teeth, and thus diminish, if not entirely dispense with the necessity for artificial substitutes." The reader will observe that following the figures 1840, and before the words The Baltimore College there is part of a sentence in italics. There was then omitted with that about a page of matter, without any sign of such omission, which should have been there in common fairness. This matter was important to the just interpretation of the passage, because it was explanatory, and showed what was considered professional dentistry, and what not—it showed that I was not unmindful of what had been done previous to 1840 ; for it was there stated that porcelain teeth were first made in France about 1820, and that Washington bought a set of teeth (1799), carved out of a solid slab of ivory. It moreover spoke well of mechanical labor, which Dr. Allen complains that I depreciate, viz : "This art (mechanic) was justly highly appreciated, because it was very useful, and gave dentists a high position in popular estimation ; it opened the way for dentistry as a profession." If this passage had been quoted, it would have been unnecessary for him to have advised me to go back 400 years B. C. to find the origin of dentistry ; for it would have been apparent that I had already gone back to a time when it was sufficiently primitive and rude.

It is astonishing that a man of Dr. Allen's intelligence should put himself to so much trouble to establish a point of no practical importance ; for, even if it is true, as he quotes from Hippocrates, that natural teeth, those made of bone or ivory were fastened to other teeth in the mouth by ligatures of flax, silk, or gold or silver wire, they would have about as much claim to be called even mechanical dentistry beside his continuous gum, as that Eve's fig leaf would to be called millinery beside the dresses for a court ball. It is well enough for an antiquary to go mousing around among old musty books, or to puzzle his head with hard Greek; because he may find something curious, or that will show that the primitive people of the earth had the same wants as those of to-day; and he may discover their first attempts at supplying them, which is a laudable undertaking for a gentleman of leisure and slight utilitarian proclivities; but for Madam Flounce, the Court milliner, to declare that Eve was the first dress maker, and as such, was entitled to full recognition by the sisterhood, would show that she is not particular as to her professional associates, whatever it might as to her judgment or taste.

The truth is, Dr. Allen is drawing it rather fine; and when he seriously prints as one to be believed, a statement like the following, it shows that he has a capacity for credulity truly Oriental: "Herodotus informs us that dentistry was practiced in Egypt as a distinct branch of surgery at that time; and corroborating evidences now exist in the fact that *good gold fillings* have been recently found in the teeth of mummies, which must have been inserted more than two thousand years ago." That gold has been found, not *good gold fillings*, in the teeth of a mummy is known to the public, as it has gone the rounds of the papers and the dental journals. A writer in the New York Dental Journal says: "We do not, however, think that implicit reliance should be placed upon the statement that, because these bodies were found with gold and aromatic preparations in the teeth, it follows that filling teeth to preserve them was customary in *life*." He suggests that it was used to preserve the teeth after death,—a part of the embalming process. He continues: "But another explanation of the reason of the Egyptians for using gold in the teeth exists in the well known fact that this metal is constantly discovered in mummies, in various forms, and applied to various uses. Thus have been found gold plates in the mouth and upon various parts of the body, and gilding the organs was a favorite mode of ornamentation, in the embalming of exalted personages. But the general practice of dentistry does not appear to have been necessitated in those days, by the condition of the teeth; for certain it is that in the mummies which have been exhumed by the many explorers of those buried cities of the dead, many evidences exist, showing the teeth to have been perfectly sound, untouched by disease at the time of death."

These extracts show how much importance is attached to the discovery of gold in the tooth of a mummy by a writer, who had no case to make out; and who did not wish to exalt ancient at the expense of modern dentistry. They moreover show that there was no great public want for dentists at that time. That some rude efforts were made to replace the teeth when lost, even in ancient times, is known to all persons versed in history. That, until about the commencement of the present century, there was very little improvement in the manner of tying natural teeth or those made of bone or ivory to the natural teeth in the mouth, from that described by Hippocrates, and already quoted, can be proved by abundant evidence. A similar notice is quoted from Cicero: De leges, ii. 24, "Cui auro dentes vincti erant;" and Martial also says:

> "Sic dentata tibi videtur ægre,
> Empti offibus Indicoque cornu."

Without multiplying quotations or authorities, it will be sufficient to state that this was the condition of what the Doctor calls dentistry to a very recent period. The next step in improvement consisted in carving solid slabs of ivory to fit the gum; when teeth were either carved upon that, or holes or sockets were drilled into it for the reception of natural teeth. Of this class were the sets of teeth made for Washington. Porcelain teeth were first made about 1820, and at about the same time plaster of Paris models came into use. Thus it will be seen that teeth, mounted upon gold plate, could not have been used prior to that, and it is probable they did not come into use for several years later. Dr. S. S. Parmly, in his Lectures on the Natural History and Management of the Teeth—N. York, 1821, p. 31, says: To obviate objections of this kind (to human teeth) I have had recourse to a safe and durable substitute, equal, by its several advantages, to the human teeth, in those of certain quadrupeds, smaller than the hippopotamus, and possessing, at the same time, a finer enamel." On the same page he says: "Artificial teeth have been formed of what has been termed mineral paste, which is a substance similar to fine porcelain." These teeth were made by M. Maury, of Paris, and were, doubtless, a great improvement on bone work, but Dr. Parmly nowhere intimates that he ever used them. All the French porcelain teeth I ever saw, previous to 1840, were very badly shaped, and they were similar in color to the common table ware of the time. Dr. Allen, after quoting the names of several writers on the natural history and the anatomy of the teeth, etc., the most of whom, after Hunter, wrote from about 1800 to 1830, says: "From these and others we have culled the basis of our profession; and Americans have done much, it is true, to elevate dental science, and a just tribute is now accorded to them by the Europeans, as well as those of our own country, for it. But to say that dentistry originated here in the United States is a false assumption, that should not be handed down to posterity in our dental records of the present era." It is true that most of our knowledge of anatomy, materia medica and therapeutics is derived from Europeans. It has been gathered there, a little by little, for several centuries, and of course it forms, with modern physiology and therapeutics, the basis of our profession. But none of these writers knew anything of the modern treatment of diseased teeth, or of dental periostitis; and it is believed that no European physician or surgeon could now pass a dental examination on periostitis of the teeth, unless he derived his knowledge from conversation with dentists or from the perusal of their works. Even Tomes' Dental Surgery, American edition, 1859, says nothing of the treatment of acute or chronic periostitis, as now practiced, but he says in substance, if the disease does not yield to leeches or some topical applications, the tooth must be extracted.

Within a few years, distinguished writers in the London Lancet have sneered at and cautioned practitioners against using arsenic for the destruction of the nerve, as a dangerous American practice. To show that American dentists early obtained a high reputation in Europe, even at the time when some of the authors quoted by Dr. Allen were writing and publishing their works, it is only necessary to say that Dr. Parmly was practicing there with success before 1820. American dentists are still the first in Europe, though no better than their brethren at home. To show the early reputation of American dentists abroad, I append the following note:

"My Dear Mrs. Roland:—A most excellent and skillful young man, recommended by Astley Cooper and Carlisle, the celebrated surgeons, is the bearer of this note to you. Mr. Montague desires me to say that if you ever want any operation on your teeth, you will be forever obliged by his pointing out this operator, for whom we have a great regard. He has put in a tooth for Mr. Montague, so skillfully as not to be detected by the nicest eye, upon an entire new principle. Any civility Mr. Roland is disposed to show Mr. Parmly, in recommending him, will be considered as an obligation by Mr. Montague.

I am truly yours, with kind regards,

London, Sept. 1, 1819. D. B. Montague."

Of Dr. Allen's next quotation a paragraph must here suffice: "At last, in 1854, a rational theory was promulgated for preventing ulceration and curing ulcerated teeth, and about the same time, as if to fix and make that the initial point for the *advent of dentistry as a profession*, several improvements were made in preparing gold for dental purposes, that enabled dentists to make much more dense and durable fillings. * * * The dental profession was then fairly established. In theory, at least, there was no longer any, or but little need of mechanics or manufacturers of artificial sets of teeth." But of these discoveries, which gladdened the hearts of all true dentists, which opened new fields for labor and usefulness, which made it possible for them to serve their fellow-beings in a nobler walk,—without mutilation, without changing the expression or individuality, without severe pain or loading the mouth with a costly and cumbersome piece of mechanism, the Doctor makes infinite merriment. He cries Eureka! and chuckles in his glee at the idea of founding a profession upon several simultaneous discoveries of means to save important organs, when diseased, which before were unsavable, and which rendered it possible for dentists to dispense with mechanical labor. His next quotation is from an article of mine, published more than a year since in the Register. "Never warrant your operations for a day; make your plugs stick, but do not warrant them to do so." Since he has called attention to this paragraph by quoting it, I will commend the rule to his serious consideration, and I hope he will adopt it in his practice. He then continues, in a strain of levity unbecoming the gravity of the subject and the momentous interests of society dependent upon the labors of professional dentists, to cast a doubt upon the efficacy of their operations; and quotes a law of England, passed in 1770; the application or apposition of which to the subject is not very apparent, unless he uses it as an authoritative proof that some teeth were lost one hundred years ago. If such was his intention, it could have been expressed in terms much more clear, pertinent and perspicacious, and he would have been believed without any endorsement. Again he quotes: "There are two classes of dentists, or rather there are dentists and mechanics, from whom very different treatment may be expected. The one, basing their practice on a knowledge of the human system and the laws that govern it, will preserve the natural teeth ; they will refuse to extract them simply because they ache or there is a gum-boil at the roots." He there makes a full period, where there was a semicolon in the text, thus making the refusal absolute, and, after omitting about a dozen lines, which contain some of the conditions on which they will extract them, viz: "*or if they do it, they will do so reluctantly for persons who persistently demand it*, and refuse to submit to the necessary treatment to preserve the teeth." A remarkable omission for a reviewer, who has hitherto complained that the article he was reviewing contained too few conditions or qualifications of statement. He then continues by quoting the first proposition of the next sentence: "The patients of the first preserve their teeth." And there ends, omitting the balance of a long sentence, and leaving it to be fairly inferred that he sought, by adroit quotation and omission, to annoy rather than to vindicate the truth, and to convict the writer he was reviewing of a contradiction, in terms. This is rendered the more probable by the question he immediately and exultantly asks: "Then why do they ache and have gum-boils?" The answer to this is ready. The patients of competent, professional dentists—those who place confidence in them and follow their directions, very seldom have toothache or gum-boils. Knowing the value of natural teeth, they have them attended to and plugged as soon as decay commences. It is their new patients, those who have listened to the promises of, and placed confidence in mechanics or incompetent operators, until they have got them into serious trouble, from which they are unable to extricate them, who present these cases for treatment. Is the answer satisfactory? The Doctor continues his quotations: "The patrons of the other get a set of shining white teeth, which every one knows to be artificial, in return for which they are but imperfectly nourished," etc. (See Register, page 468,) and adds:

"Although there may be some to whom the above remarks may be applied with truth, yet, to speak thus disparagingly of all who insert artificial dentures, evinces one of two things: either that the position that he occupies in the profession is unfavorable to command a full view of it; or his zeal to eulogise one branch of dentistry at the expense of another, has warped his better judgment." In reply to this, I have but to say: No true dentist, who desires or labors for the elevation of the profession, and the best interests of his patients, although artificial teeth are made in his office, or he makes them himself, will feel in any way compromised or aggrieved by the above paragraph. While conscientiously discharging his duty to his patrons, he will regret the necessity of making them, and that people will be so careless or infatuated as to require it; but he will feel that neither he nor the profession are in any way injured or disrespectfully used by any man, who habitually and studiously labors to convince people of the value of their teeth, to induce them to preserve them and avoid artificial ones. If the conduct there described does not apply to him, he will rejoice that it was written.

The difference between Dr. Allen and myself is one of definition. I define dentistry as a profession to consist in chair operations and the employment of such medicinal means, whether internal or topical, as are adapted to preserve the health or teeth, or to restore them when impaired. He gives it a much greater latitude.

Can it be possible the hypersensitiveness manifested by Dr. Allen can be due to his long continued study and experiments to improve continuous gum; and that he is verging on that psychological condition, often met with in inventors and persons who have taken out a patent, described by two Greek words, which, if his reading of Greek literature, to ascertain the ancient status of dentistry, has been as laborious and exhaustive as inferred from his review, he must be familiar with; they are *monos* and *mania*.

A word, and I am done. I exceedingly regret that Dr.

Allen has felt called upon to write a review, which has placed me in this relation to him. I, too, have known him for many years, and I sincerely respect him for the long continued study and labor he has devoted to perfect an artificial denture that is really beautiful and often valuable. I know him to be a good mechanic, and have no reason to doubt his ability as an operator.

